# Effects of activity participation and cognitive levels on depression in middle-aged and older adults with chronic illness: a national cross-sectional study

**DOI:** 10.3389/fpsyg.2024.1415715

**Published:** 2024-10-14

**Authors:** Lei Jin, Feiyue Jing

**Affiliations:** College of Physical Education and Health, Guangxi Normal University, Guilin, Guangxi, China

**Keywords:** middle-aged and older adults, chronic illness, activity participation, cognitive level, depression

## Abstract

**Introduction:**

The world population is rapidly aging, and depression mainly affects middle-aged and older adults with chronic diseases and cognitive impairments. The sample for this study was obtained from the China Health and Retirement Longitudinal Study (CHARLS) public database. The sample size for inclusion was 12,767. There were 6,773 females and 5,994 males, with an overall low level of education. This study aims to provide a theoretical and practical reference basis for the clinical non-pharmacological treatment of depression in middle-aged and older adults (age ≥ 50 years) with chronic diseases. Additionally, the study seeks to promote the development of mental health interventions for middle-aged and older adults (age ≥ 50 years) with chronic diseases, ultimately enhancing the sense of well-being and quality of life for this demographic.

**Methods:**

Cognitive functioning and depressive symptoms of the study participants were assessed using the Mini-Mental State Examination Scale (MMSE) and the short version of the Center for Epidemiological Studies Depression Scale (CESD-10).

**Results and discussion:**

Binary logistic regression results showed that among middle-aged and older adults (age ≥ 50 years) with chronic diseases, participation in physical activity [OR = 1.397; 95% CI (1.181–1.651); *p* < 0.05] was more effective than participation in social activities [OR = 0.997; 95% CI (0.924–1.076); *p* < 0.05] for preventing depression. Those with cognitive impairment [OR = 1.206; 95% CI (1.089–1.335); *p* < 0.05] were more likely to experience depression than those without cognitive impairment. Activity participation (physical activity and social activity) had a more significant effect on mild and moderate depression compared to no depression, and cognitive level had a more pronounced effect on moderate depression [OR = 1.491; 95% CI (1.278–1.740); *p* < 0.05] and major depression [OR = 2.231; 95% CI (1.282–3.884); *p* < 0.05]. Within the specific cohort of middle-aged and older adults (age ≥ 50 years) with chronic diseases, both activity participation and cognitive level exert a significant influence on the prevention and intervention of depression. Engagement in physical activity, participation in social activities, and enhanced cognitive functioning emerged as protective factors against depression. Therefore, the policy-maker should strengthen the prevention and treatment of depression in a comprehensive manner through the promotion of physical and social activities and the enhancement of cognitive level, so as to safeguard the mental health of middle-aged and older adults with chronic diseases.

## 1 Introduction

The world’s population is aging rapidly, with 1 billion people aged 60 years or older globally in 2020. By 2030, this figure is projected to rise to 1.4 billion, constituting one-sixth of the global population. Furthermore, by 2050, it is expected to reach 2.1 billion. Middle-aged and older adults (age ≥ 50 years) play a crucial role in families, communities, and society at large; however, many of them grapple with chronic diseases [World Health Organization (WHO), 2023a]. However, in China, according to the China Health and Retirement Longitudinal Study (CHARLS, 2020) data statistics, the percentage of middle-aged and older adults with chronic diseases is alarmingly high at 65.92%. Moreover, a significant portion of this population may experience mobility issues, chronic pain, cognitive impairment, or other health-related challenges. As individuals age, approximately 14% of adults aged 60 and older contend with mental disorders, accounting for 10.6% of the total disability life expectancy within this age group. Notably, depression stands out as the most prevalent mental health issue (Institute of Health Metrics and Evaluation, 2023). According to CHARLS (2020) data, the percentage of middle-aged and older adults with chronic diseases who also experience depression is notably high, reaching 61.3%.

Depression is a mental disorder characterized by persistent and significant low mood, with complex and varied pathological mechanisms. Clinically, patients usually present with prolonged sadness, despair, reduced sense of self-worth, and loss of interest in daily activities. This is accompanied by difficulty concentrating, decision-making, memory loss, fatigue, changes in appetite (leading to significant changes in body weight), sleep disturbances (insomnia or hypersomnia), and even suicidal thoughts and behaviors (Devita et al., 2022). Individuals who have undergone adverse life events, such as illness, unemployment, bereavement, or traumatic experiences, are more susceptible to developing depression (Kendler, 2020). The correlation between depression and physical health is substantial, with depression emerging as a common comorbidity in various chronic diseases, including cancer, cardiovascular, metabolic, inflammatory, and neurological conditions (Gold et al., 2020). Research findings indicate associations between depression and myocardial infarction (MI), stroke, and atrial fibrillation (AF) (Li et al., 2022). Major depression (MDD) has also been linked to increased odds of coronary artery disease (Sui et al., 2023), and cancer patients exhibit a fourfold higher prevalence of depression than the general population (Bortolato et al., 2017). Furthermore, individuals with multiple chronic diseases face a threefold higher risk of developing depression than their healthy counterparts. The odds of developing depression increase by 45% for each additional chronic disease (Read et al., 2017). Many factors influencing depression, such as a lack of physical activity or cognitive impairment, are also recognized risk factors for chronic diseases such as cardiovascular disease, cancer, diabetes, and respiratory diseases. Individuals with these conditions may find themselves grappling with depression due to the challenges associated with managing their health (Devita et al., 2022). The World Health Organization acknowledges the effectiveness of activity engagement and cognitive behavioral therapy in preventing depression in middle-aged and older adults [World Health Organization (WHO), 2023b].

Activity participation encompasses engagement in physical activities (such as sports or exercise) and social activities (including socializing with friends or participating in various clubs) (Tcymbal et al., 2022). There exists an inverse curvilinear dose–response relationship between physical activity and depression, with the gradient of the relationship steepening as activity decreases. Notably, there is significant heterogeneity (Pearce et al., 2022). Physical activity has demonstrated efficacy in treating mild to moderate depression and reducing mortality and symptoms of major depression (Chen et al., 2022). The primary biological mechanism through which exercise mitigates depression involves the stimulation of several neuroregenerative processes associated with depression, such as the hippocampus, brain-derived neurotrophic factor (BDNF), and dopamine (Kandola et al., 2019). Additionally, studies by Paolucci et al. (2018), Schuch et al. (2014), and Heijnen et al. (2016) collectively suggest that exercise reduces inflammation and enhances resistance to oxidative and physiological stress, and that an understanding of these mechanisms may enhance the design of exercise interventions to optimize therapeutic response and reduce depressive symptoms. The higher the level of social activity, the lower the risk of depression (Ryu et al., 2023), and attending social gatherings with friends and neighbors can reduce depressive symptoms (Min et al., 2016). The studies by Lagunes-Córdoba et al. (2022), Stavrova and Ren (2023), collectively indicate that active participation in socializing can reduce or prevent depression through three mechanisms: engaging in social activities can provide emotional support, a platform to share problems and feelings, and alleviate feelings of loneliness, fostering a sense of understanding, acceptance, and care, thus reducing psychological burdens. Establishing positive social relationships can boost an individual’s self-esteem, and acceptance and recognition by the community can enhance the sense of self-worth (Lee et al., 2023). Participation in activities such as group activities or volunteering can infuse life with purpose and a sense of fulfillment, thereby aiding in alleviating depression (Stevens et al., 2021).

There exists a relationship between cognitive level and depression, though it is not a simple and direct causal connection (Halahakoon et al., 2019). Cognitive level refers to an individual’s capacity to process, comprehend, and assess information, whereas depression is a mood disorder characterized by persistent and severe frustration (Clark and Beck, 2010). Individuals with low cognitive levels are prone to negative cognitive bias, entailing negative interpretations and evaluations of things and situations. This negative thinking may render them more susceptible to depression (Beevers et al., 2019). Conversely, individuals with high cognitive levels typically possess enhanced problem-solving and adaptive skills, an understanding and recognition of their own emotions and needs, and an increased ability to cope with life’s challenges, thus reducing the risk of depression (Gold and Otte, 2019).

In individuals without depression, the psychological impact of physical activity, social activities, and cognitive functioning is less pronounced compared to those with depressive disorders. This difference stems from the higher emotional baseline, more balanced neurophysiological states, and stronger self-regulatory mechanisms in non-depressed individuals (Tarazona-Santabalbina et al., 2016). Additionally, their stable social support networks and effective cognitive coping strategies reduce the need for external interventions to regulate emotions (Kim et al., 2017). Consequently, while physical activity, social interactions, and cognitive health remain beneficial, the emotional and psychological shifts they induce are subtler, lacking the profound effects observed in individuals with depression, who often rely more heavily on these factors for emotional well-being (Vance et al., 2016).

In summary, prior domestic and international research has established a robust theoretical foundation for this paper and has provided objective and scientific support in variable selection. Among middle-aged and older adults, depression predominantly impacts individuals with chronic illnesses and cognitive impairments, leading to distress, family disruption, disability, deteriorating treatment outcomes for various diseases, and an increased mortality risk (Alexopoulos, 2005). However, certain limitations exist in previous studies. Firstly, with the accelerated aging process, many chronic diseases and depression are gradually affecting a “younger” demographic. Few studies have specifically focused on middle-aged and older adults with chronic diseases as a distinct group, validating the relationship between depression and physical activity in this population. Most studies have primarily explored the connection between chronic diseases and depression. Secondly, many studies have concentrated on a single chronic illness and a singular form of activity engagement (e.g., physical or social activity) when investigating depression. This approach overlooks the shared impact of activity engagement on depression in patients with chronic illnesses as a whole. Additionally, most studies have solely considered cognitive level as a mediating factor, with few exploring depression in middle-aged and older adults with chronic illnesses by placing it on the same dimension (explanatory variable) as activity participation. Building upon these observations, this paper proposes the following three research hypotheses:

*H1*: Among middle-aged and older adults with chronic diseases, activity participation mitigates the risk of depression and proves to be more efficacious for physical activity than for social activities.

*H2*: Among middle-aged and older adults with chronic diseases, higher cognitive levels are associated with a lower risk of depression.

*H3*: Among middle-aged and older adults with chronic diseases, activity participation (physical activity and social activity) exerts a more significant effect on mild and moderate depression, while cognitive level exerts a more significant effect on moderate and severe depression, in comparison to individuals with no depression.

## 2 Methods

### 2.1 Sample and data sources

The data for this study are derived from the latest dataset (CHARLS, 2020) of the China Health and Retirement Longitudinal Study (CHARLS), released on November 16, 2023. CHARLS aims to collect a high-quality microdata set representative of Chinese middle-aged and older adults aged 45 years and older at the household and individual levels. This dataset is used to analyze China’s population aging and foster interdisciplinary research on aging. The national baseline survey was conducted in 2011, covering 17,000 people in approximately 10,000 households across 150 county-level units and 450 village-level units. These samples were subsequently tracked every 2–3 years, and the data were made available to the academic community 1 year after the survey was completed (CHARLS, 2020).

The population for this study consisted of middle-aged and older adults with chronic diseases (*N* = 12,767), and the sample inclusion criteria were as follows: (1) age greater than or equal to 50 years old; (2) a positive response to question Da003 in the questionnaire (Has a doctor ever told the respondent that he or she has a chronic disease?) or Da004 (Does the respondent know he/she has a chronic disease?). Responses of “yes” were categorized as having a chronic disease; and (3) all missing values for the included variables were eliminated.

### 2.2 Selection of variables

#### 2.2.1 Dependent variables

The depression variable was computed using the Center for Epidemiologic Studies Depression Scale (CES-D) provided in the questionnaire. The CES-D is widely recognized by international scholars and extensively used in the field of mental health (Chen and Mui, 2014). CHARLS designed 10 relevant questions in the questionnaire to inquire whether respondents have specific feelings. The sum of the scores for these 10 questions represents the respondent’s CES-D score, which is inversely proportional to the level of mental health; the higher the score, the higher the respondent’s level of mental depression, indicating worse mental health. For this study, the total CES-D score was defined as 30 (0 for answering few or none of the questions; 1 for not too many; 2 for sometimes or half the time; and 3 for most of the time). Consequently, this led to a total score of less than or equal to 5 indicating no depression (assignment: “0”), and greater than 5 indicating the presence of depression (assignment: “1”) for dependent variable 1 (Depression or not). For dependent variable 2 (Depression level), a total score of less than or equal to 5 denotes no depression (Category A, assignment: “0”), 6–15 indicates mild depression (Category B, assignment: “1”), 16–25 suggests moderate depression (Category C, assignment: “2”), and 26–30 indicates major depression (Category D, assignment: “3”).

#### 2.2.2 Independent variables

Dependent Variable 1 (Physical Activity Participation) is derived from CHARLS questionnaire question DA032, which inquires about the respondent’s engagement in various types of physical activity, including high-intensity exercise, moderate-intensity exercise, and low-intensity exercise. The question prompts respondents to recall activities in which they exercised for at least 10 min on each occasion and whether they consistently engage in such activities for at least 10 min each week. Respondents who indicated the type of physical activity were considered to be participating in physical activity (assignment: “1”), while those who did not were considered not participating in physical activity (assignment: “0”). Additionally, participation in high-intensity activity, moderate-intensity activity, and low-intensity activity were treated as independent variables 1–1, independent variables 1–2, and independent variables 1–3, respectively. Non-participation was assigned the value of “0,” and participation was assigned the value of “1.” The CHARLS questionnaire defines very physically strenuous and intense activities (high-intensity activity) as activities that induce rapid breathing, such as lifting heavy objects, digging, plowing, aerobics, fast biking, and carrying loads on a bike. Moderately physically strenuous activities (moderate-intensity activity) are described as activities that make one breathe a little faster than usual, such as lifting something light, bicycling at regular speed, mopping, doing tai chi, and walking briskly. Light physical activity (low-intensity activity) primarily includes walking, encompassing movement from place to place at work or at home, as well as walks for recreation, sport, exercise, or entertainment.

Dependent variable 2 (Social Activity Participation) is derived from CHARLS questionnaire question DA038, which queries respondents about their engagement in various social activities over the past month. The activities listed include visiting neighbors, socializing with friends, playing mahjong, chess, or cards, attending community events, offering assistance to non-cohabiting relatives, friends, or neighbors, participating in activities like dancing, working out, practicing qigong, engaging in community organization events, volunteering, participating in charitable activities, caring for sick or disabled non-cohabiting individuals, attending school or training courses, or other specified social activities. Respondents have the option to select multiple responses. Respondents are considered to be involved in social activities if they answer affirmatively to any of the listed social activities (assignment: “1”). Conversely, they are considered not involved in social activities if they answer “none of the above” (assignment: “0”).

Dependent Variable 3 (Cognitive Impairment) was assessed using the Mini-Mental State Examination (MMSE) based on respondents’ answers to the CHARLS questionnaire. The MMSE comprises a total of 41 items across four categories of cognitive functioning dimensions. The total score ranges from 0 to 30, with a lower score indicating poor cognitive functioning. The test scores are closely related to the highest level of education. Normal cut-off values for cognitive impairment are as follows: illiterate >17 points, elementary school >20 points, secondary school and above >24 points, indicating no cognitive impairment (assignment: “0”). Conversely, scores below these thresholds suggest cognitive impairment (assignment: “1”).

#### 2.2.3 Control variables

The control variables encompass basic demographic indicators, including gender, age, highest level of education, household type, and marital status. Sex is derived from questionnaire question BA001, where the interviewer records the respondent’s sex as male or female. Male is assigned as “1,” and female is assigned as “0.” Age is derived from questionnaire question BA003, which captures the respondent’s real date of birth. Those aged 50–60 are assigned as “0,” 61–70 as “1,” 71–80 as “2,” and those older than 80 as “3.” The highest level of education is determined by questionnaire question BA010, inquiring about the respondent’s current educational attainment. Illiteracy is assigned as “0,” elementary school as “1,” secondary school as “2,” polytechnic as “3,” and a bachelor’s degree or above as “4.” Household type, as per questionnaire question BA009, designates the current household type of the respondents. Agricultural is assigned as “0,” and non-agricultural (urban) is assigned as “1.” Marital status, derived from questionnaire question BA011, represents the respondent’s current marital status. Other statuses (divorced, widowed, unmarried) are assigned “0,” while being married is assigned “1.”

### 2.3 Model setting

#### 2.3.1 Binary logistic regression modeling

This study was modeled and analyzed using SPSS.27 software, with the dependent variable 1 (presence or absence of depression) set as a binary outcome. The criterion of “CES-D total score less than or equal to 5 or greater than 5” was used to categorize individuals as having no depression (0) or having depression (1). A binary logistic regression model was selected, represented by Equation 1:


(1) $$Ln\left[\frac{p\left(Y=1\right)}{1-p\left(Y=1\right)}\right]=\beta_{0}+\overset{k}{\underset{i=1}{\sum }}\beta_{i}X_{i}$$

Where “*Y* = 1” is defined if one suffers from depression, and “*Y* = 0” is defined if one does not suffer from depression. *X_i_* represents the i-th explanatory variable influencing depression, where *K* is the number of explanatory variables. *β_i_* denotes the regression coefficient of the explanatory variable *X_i_*, indicating the correlation with the presence or absence of depression. *β_0_* is the intercept term. The probability of experiencing depression is expressed as in Equation 2:


(2) $$p\left(Y=1|X\right)=\frac{\exp\left(\beta_{0}+\sum_{i=1}^{k}\beta_{i}X_{i}\right)}{1+\exp\left(\beta_{0}+\sum_{i=1}^{k}\beta_{i}X_{i}\right)}$$

The control variables were incorporated into the model expression (1) to formulate Model 1. Building on Model 1, the control variables underwent dummy-variable treatment, and independent variable 1 (physical activity participation), independent variable 1–1 (high-intensity activity), independent variable 1–2 (moderate-intensity activity), independent variable 1–3 (low-intensity activity), independent variable 2 (social activity participation), and independent variable 3 (cognitive impairment) were introduced to develop Model 2. Both Model 1 and Model 2 utilized a forced input model. A statistically significant level of *α* < 0.05 (95% CI) was employed for the output.

#### 2.3.2 Multivariate logistic regression modeling

In this study, SPSS.27 software was utilized for modeling and analysis. The dependent variable 2 (depression level) was categorized into four levels, with a total score of less than or equal to 5 indicating no depression (assigned value: “0”), 6–15 indicating mild depression (assigned value: “1”), 16–25 indicating moderate depression (assigned value: “2”), and 26–30 indicating severe depression (assigned value: “3”). The multiple logistic regression model Equation 3 was selected:


(3) $$Ln\left(\frac{P_{j}}{P_{J}}\right)=\beta_{0}+\overset{p}{\underset{i=1}{\sum }}\beta_{i}x_{i}$$

Where *P_j_* is the probability that the explanatory variable is in category *j*, *PJ* is the probability that the explanatory variable is in category *J (J ≠ j)*, and category *J* is the reference group. *Ln (P_j_/PJ)* represents the generalized Logit *P*, which is the natural logarithm of the ratio of the two probabilities. The dependent variable 2 in this study has four categories, namely A, B, C, and D. Consequently, category A (no depression, assigned value: “0”) is employed as the reference category to construct three logistic regression models.

Type B model (Equation 3-1):


(3-1) $$\mathrm{Logit}P_{B}=Ln\left[\frac{P\left(y=B|X\right)}{P\left(y=A|X\right)}\right]=\beta_{0}^{B}+\overset{P}{\underset{i=1}{\sum }}\beta_{i}^{B}X_{i}$$

Type C model (Equation 3-2):


(3-2) $$\mathrm{Logit}P_{C}=Ln\left[\frac{P\left(y=C|X\right)}{P\left(y=A|X\right)}\right]=\beta_{0}^{C}+\overset{P}{\underset{i=1}{\sum }}\beta_{i}^{C}X_{i}$$

Type D model (Equation 3-3):


(3-3) $$\mathrm{Logit}P_{D}=Ln\left[\frac{P\left(y=D|X\right)}{P\left(y=A|X\right)}\right]=\beta_{0}^{D}+\overset{P}{\underset{i=1}{\sum }}\beta_{i}^{D}X_{i}$$

In the above equations, *β_0_* is the threshold value; the regression coefficient *β_i_* indicates the average amount of change in logarithmic odds or Logit *P_j_* when a certain independent variable changes by one unit. The Odds Ratio (OR), representing the magnitude of the change in OR caused by a one-unit change in any of the independent variables, can be directly interpreted through logistic regression. The OR can be considered an effect indicator, signifying the degree of influence of the independent variable on the odds of the corresponding dependent variable (Wang et al., 2019). Since the impact of control variables on depression has been investigated in the binary logistic regression model 1, only the independent variables were included in model 3. The level of statistical significance used for the output was *α* < 0.05 (95% CI).

## 3 Results

### 3.1 Reliability and validity tests of the CES-D and MMSE scales

In this study, the reliability and validity of the CES-D and MMSE scales were assessed using confidence statistics and factor analysis. The Cronbach’s α value of the CES-D scale was 0.797, exceeding 0.7, and the KMO value was 0.881 (*p* < 0.001), surpassing 0.8. These results indicated that the scale could be effectively used to evaluate whether middle-aged and older adults with chronic diseases experience depressive symptoms. For the MMSE scale, the Cronbach’s alpha value was 0.744, surpassing 0.7, and the KMO value was 0.784 (*p* < 0.001), exceeding 0.7 and approaching 0.8. These findings suggest that the scale can be utilized to assess whether middle-aged and older adults with chronic diseases suffer from cognitive disorders, demonstrating high reliability and good validity. All parameters of the reliability test for the CES-D and MMSE scales are presented in Table 1.

**Table 1:** CES-D & MMSE scale reliability and validity test.

Scale name	Test methods	Parameter name	Parameter	
CES-D	Reliability statistics	Cronbach’s Alpha	0.797	
		Cronbach’s Alpha Based on Standardized Items	0.806	
		*N* of Items	10	
	Factor analysis (KMO and Bartlett‘s Test)	Kaiser-Meyer-Olkin measure of sampling adequacy	0.881	
		Bartlett’s Test of Sphericity	Approx Chi-Square	29593.507
			Df	45
			Sig.	0.000
MMSE	Reliability Statistics	Cronbach’s Alpha	0.744	
		Cronbach’s Alpha Based on Standardized Items	0.773	
		*N* of Items	41	
	Factor analysis (KMO and Bartlett‘s Test)	Kaiser-Meyer-Olkin measure of sampling adequacy	0.784	
		Bartlett’s Test of Sphericity	Approx Chi-Square	67672.966
			Df	820
			Sig.	0.000

Significance tests are standardized: sig < 0.05 (95% Cl).

### 3.2 Descriptive statistical analysis of variables

Among middle-aged and older adults with chronic diseases (*N* = 12,767), 7,830 individuals (61.3%) were identified as suffering from depression. The majority experienced mild or moderate depression, with 5,592 (43.8%) having mild depression and 2,068 (16.2%) having moderate depression. Regarding the form of activity participation, physical activities were more prevalent than social activities among middle-aged and older adults with chronic diseases, with 87.8% participating in physical activities and only 46.8% participating in social activities. In terms of cognition, the overall cognitive level of middle-aged and older adults with chronic diseases was low, and 10,784 individuals (84.4%) were identified as having cognitive impairment. Detailed categorization, assignment, and parameters of the variables are presented in Table 2. However, for the correlation of the independent and control variables with the dependent variable 2 (level of depression), this study employed cross-tabulation and chi-square tests. The chi-square values for independent variable 1, independent variable 1–1, independent variable 1–2, independent variable 1–3, independent variable 2, independent variable 3, and dependent variable 2 (degree of depression) were 28.009, 66.529, 44.239, 17.467, 14.740, and 33.493, respectively, with *p* values all less than 0.05. The chi-square values for control variables (age, gender, household type, highest education level, marital status) and dependent variable 2 (degree of depression) were 167.642, 373.386, 240.510, 366.367, 53.173, respectively, with p values less than 0.01. There is a strong correlation between the dependent variable 2 and all the independent variables and control variables. The cross-frequency of independent and control variables on dependent variable 2 and related detailed parameters are shown in Table 3.

**Table 2:** Descriptive statistics for variables(*N* = 12,767).

Variable	Categorization	Assignment	Frequency	Percentages (%)
Age	50–60	0	5,041	39.48
	61–70	1	4,922	38.55
	71–80	2	2,200	17.23
	Above 80	3	604	4.74
Gender	Female	0	6,773	53.05
	Male	1	5,994	46.95
Household type	Agricultural	0	10,553	82.66
	Urban	1	2,214	17.34
Highest level of education	Illiteracy	0	5,977	46.82
	Elementary school	1	6,320	49.51
	Secondary school	2	258	2.02
	Polytechnic	3	142	1.11
	Bachelor’s degree or above	4	70	0.54
Marital status	Other statuses (divorced, widowed, unmarried)	0	2,285	17.90
	Married	1	10,482	82.10
Depression or not	No	0	4,937	38.67
	Yes	1	7,830	61.33
Depression level	No depression (Category A)	0	4,937	38.67
	Mild depression (Category B)	1	5,592	43.80
	Moderate depression (Category C)	2	2068	16.20
	Major depression (Category D)	3	170	1.33
Physical activity participation (X_1_)	No	0	1,556	12.19
	Yes	1	11,211	87.81
High-intensity activity (X_1-1_)	No	0	8,331	65.25
	Yes	1	4,436	34.75
Moderate-intensity activity (X_1-2_)	No	0	5,936	46.49
	Yes	1	6,831	53.51
Low-intensity activity (X_1-3_)	No	0	3,057	23.94
	Yes	1	9,710	76.06
Social activity participation (X_2_)	No	0	6,788	53.17
	Yes	1	5,979	46.83
Cognitive impairment (X_3_)	No	0	1983	15.53
	Yes	1	10,784	84.47

Variable assignment (“0,” “1,” “2,” “3,” “4”) only indicates the classification, no practical significance.

**Table 3:** Controlled comparison of different levels of depression (*N* = 12,767).

Variable	Assignment	*N* (%)	No depression (*N* = 4,937)	Mild depression (*N* = 5,592)	Moderate depression (*N* = 2068)	Major depression (*N* = 170)	*X*^2^ value	*p*-value (2-sided)
Age	0	5,041 (39.48%)	2005 (40.61%)	2,204 (39.41%)	759 (36.70%)	73 (42.94%)	167.642	<0.001
	1	4,922 (38.55%)	1755 (35.55%)	2,232 (39.91%)	868 (41.97%)	67 (39.42%)		
	2	2,200 (17.23%)	807 (16.35%)	971 (17.37%)	395 (19.11%)	27 (15.88%)		
	3	604 (4.74%)	370 (7.49%)	185 (3.31)	46 (2.22%)	3 (1.76%)		
Gender	0	6,773 (53.05%)	2,167 (43.89%)	3,088 (55.22%)	1,399 (67.65%)	119 (70.00%)	373.386	<0.001
	1	5,994 (46.95%)	2,770 (56.11%)	2,504 (44.78%)	669 (32.35%)	51 (30.00%)		
Household type	0	10,553 (82.66%)	3,791 (76.79%)	4,727 (84.53%)	1875 (90.67%)	160 (94.12%)	240.510	<0.001
	1	2,214 (17.34%)	1,146 (23.21%)	865 (15.47%)	193 (9.33%)	10 (5.88%)		
Highest level of education	0	5,977 (46.82%)	1915 (38.79%)	2,721 (48.66%)	1,235 (59.72%)	106 (62.35%)	366.367	<0.001
	1	6,320 (49.51%)	2,728 (55.26%)	2,716 (48.57%)	813 (39.32%)	63 (37.06%)		
	2	258 (2.02%)	165 (3.34%)	83 (1.48%)	10 (0.48%)	0 (0)		
	3	142 (1.11%)	85 (1.72%)	51 (0.91%)	5 (0.24%)	1 (0.59%)		
	4	70 (0.54%)	44 (0.89%)	21 (0.38%)	5 (0.24%)	0 (0)		
Marital status	0	2,285 (17.90%)	813 (16.47%)	952 (17.02%)	480 (23.21%)	40 (23.53%)	53.173	<0.001
	1	10,482 (82.10%)	4,124 (83.53%)	4,640 (82.98%)	1,588 (76.79%)	130 (76.47%)		
Physical activity participation (X_1_)	0	1,556 (12.19%)	678 (13.73%)	586 (10.48%)	268 (12.96%)	24 (14.12%)	28.009	<0.001
	1	11,211 (87.81%)	4,259 (86.27%)	5,006 (89.52%)	1800 (87.04%)	146 (85.88%)		
High-intensity activity (X_1-1_)	0	8,331 (65.25%)	3,434 (69.56%)	3,512 (62.80%)	1,277 (61.75%)	108 (63.53%)	66.529	<0.001
	1	4,436 (34.75%)	1,503 (30.44%)	2080 (37.20%)	791 (38.25%)	62 (36.47%)		
Moderate-intensity activity (X_1-2_)	0	5,936 (46.49%)	2,477 (50.17%)	2,468 (44.13%)	920 (44.49%)	71 (41.76%)	44.239	<0.001
	1	6,831 (53.51%)	2,460 (49.83%)	3,124 (55.87%)	1,148 (55.51%)	99 (58.24%)		
Low-intensity activity (X_1-3_)	0	3,057 (23.94%)	1,148 (23.25%)	1,299 (23.23%)	558 (26.98%)	52 (30.59%)	17.467	<0.001
	1	9,710 (76.06%)	3,789 (76.75%)	4,293 (76.77%)	1,510 (73.02%)	118 (69.41%)		
Social activity participation (X_2_)	0	6,788 (53.17%)	2,599 (52.64%)	2,923 (52.27%)	1,161 (56.14%)	105 (61.76%)	14.740	0.002
	1	5,979 (46.83%)	2,338 (47.36%)	2,669 (47.73%)	907 (43.86%)	65 (38.24%)		
Cognitive impairment (X_3_)	0	1983 (15.53%)	816 (16.53%)	907 (16.22%)	246 (11.90%)	14 (8.24%)	33.493	<0.001
	1	10,784 (84.47%)	4,121 (83.47%)	4,685 (83.78%)	1822 (88.10%)	156 (91.76%)		

Significance tests are standardized: *p* < 0.05 (95% Cl).

In this study, before establishing the Logistic regression model, it is considered that multicollinearity among the independent variables may impact the prediction results. Therefore, the diagnosis of multicollinearity was performed on all independent variables, conditional on controlling for the control variables. The results indicate that the variance inflation factor (VIF) value for all independent variables is less than 5, and the tolerance value is greater than 0.1. This allows us to conclude that there is no issue of multicollinearity among the respective variables. Detailed parameters are shown in Table 4. Based on the above analysis and test, this paper establishes the binary Logistic regression model (Model 1, Model 2) and the multivariate Logistic regression model (Model 3).

**Table 4:** Independent variable multicollinearity test.

Dependent variable	Independent variable	SE	Standardized coefficients β	*t*	*p*-value	Tolerance	VIF
Depression or not	Constant	0.017		32.071	0.000		
	X_1_	0.019	0.045	3.441	0.001	0.459	2.177
	X_2_	0.009	−0.015	−1.669	0.095	0.957	1.045
	X_3_	0.012	0.027	2.999	0.003	0.987	1.013
	X_1-1_	0.010	0.055	5.904	0.000	0.890	1.123
	X_1-2_	0.010	0.042	4.261	0.000	0.803	1.245
	X_1-3_	0.014	−0.052	−4.352	0.000	0.543	1.841
Depression level	Constant	0.026		26.879	0.000		
	X_1_	0.030	0.031	2.356	0.018	0.459	2.177
	X_2_	0.014	−0.027	−2.989	0.003	0.957	1.045
	X_3_	0.018	0.046	5.162	0.000	0.987	1.013
	X_1-1_	0.015	0.052	5.559	0.000	0.890	1.123
	X_1-2_	0.015	0.044	4.504	0.000	0.803	1.245
	X_1-3_	0.021	−0.056	−4.724	0.000	0.543	1.841

Test criteria: VIF < 5, Tolerance > 0.1, *p* < 0.05 (95% Cl).

### 3.3 Overall model test results

In the test results of Model 1 and Model 2, the prediction accuracy for experiencing depression was 100 and 89.3% in middle-aged and older adults with chronic diseases. The model coefficients’ Omnibus test chi-square values were 682.587 and 762.247, with *p*-values less than 0.05. The chi-square values of the Hosmer and Lemeshow Test were 17.733 (*p* = 0.053) and 18.415 (*p* = 0.058), with *p*-values greater than 0.05. In the resultant test of Model 3, the −2 Log likelihood value is 551.165, and the goodness-of-fit and goodness-of-fit information chi-square values are 123.542 and 180.319, respectively, with *p*-values all less than 0.05. In summary, for Model 1, Model 2, and Model 3, with correct model selection, high goodness of fit, good significance, and statistical significance, they can be used for further statistical analysis of the results. The detailed parameters of Model 1 and Model 2 are shown in Table 5, and those of Model 3 are shown in Table 6.

**Table 5:** Overall test results of the binary logistic regression model.

	Predictive accuracy(%)			Omnibus tests of model coefficients			Hosmer and Lemeshow test		
	Depression	No depression	Total	Chi-square	Df	*p-*value	Chi-square	Df	*p-*value
Model 1	100	0	61.3	682.587	10	0.000	17.733	8	0.053
Model 2	89.3	24.5	64.2	762.247	16	0.000	18.415	8	0.058

Omnibus tests of model coefficients significance test criterion: *p* < 0.05 (95% Cl); Hosmer and Lemeshow Test significance test criterion: *p* > 0.05 (95% Cl).

**Table 6:** Overall test results of the multivariate logistic regression model.

	Goodness-of-fit (Gofit)			Model fitting information			
	Chi-square	Df	*p-*value	−2 Log likelihood	Chi-square	Df	*p*-value
Model 3	123.542	75	0.000	551.165	180.319	18	0.000

Significance tests are standardized: *p* < 0.05 (95% Cl).

### 3.4 Binary logistic regression results

In the Model 1, the reference group for all variables is Category 1 (Assignment: “0”). Overall, the control variables (age, gender, type of household, highest level of education, and marital status) all had a statistically significant effect on the dependent variable 1 (whether or not they had depression), with *p*-values less than 0.05. Although the *p*-value for age (2) was greater than 0.05, it did not impact the overall age significance (*p* < 0.05). It is worth mentioning that the regression coefficients for the variables of gender (1, female), type of household (1, urban), highest level of education (≠0, non-literate), and marital status (1, married) were negative and negatively correlated with having depression. Therefore, when exploring the effects of activity participation and cognitive level on depression in middle-aged and older adults, this study incorporated the above variables as control variables. On the basis of Model 1, the control variables were dummy variables, and the core independent variables were added to establish Model 2.

In the Model 2, independent variable 1, independent variable 1–1, independent variable 1–2, independent variable 1–3, independent variable 2, and independent variable 3 had a significant effect on dependent variable 1 (whether or not suffering from depression), with p-values less than 0.05, which is statistically significant. The regression coefficients of independent variable 1, independent variable 2, and dependent variable 1 (whether suffering from depression or not) are negative, i.e., activity participation and suffering from depression are negatively correlated, and physical activity participation [OR = 1.397; 95%Cl (1.181–1.651)] or social activity participation [OR = 0.997; 95%Cl (0.924–1.076)] may prevent depression. In middle-aged and older adults with chronic diseases, the odds of developing depression among those who do not engage in physical activity are 1.397 times higher than among those who do engage. Similarly, the odds of developing depression for individuals who do not participate in social activities are 0.997 times those who do participate. However, at the level of activity intensity, low-intensity activity was negatively associated with having depression [OR = 0.829; 95% Cl (0.734–0.935)]. Whereas, moderate-intensity and high-intensity activity were positively associated with having depression, i.e., participation in moderate-intensity activity [OR = 1.100; 95%Cl (1.012–1.196)] and high-intensity activity [OR = 1.215; 95%Cl (1.116–1.323)] led to depression in middle-aged and older adults with chronic diseases. At the cognitive level, there was a significant relationship between cognitive level and depression in middle-aged and older adults with chronic diseases (*p* < 0.05), suffering from cognitive impairment led to depression [*β* > 0, OR = 1.206; 95%Cl (1.089–1.335)], and the probability of suffering from depression increased by 20.6% for each unit of elevation of cognitive impairment. Detailed parameters for Model 1 and Model 2 are in Table 7.

**Table 7:** Binary logistic regression results (*N* = 12,767).

	Variable names	β	S.E.	OR (95% Cl)	*p*-value
Model 1	Constant	1.091	0.062	2.977	0.000
	Age (1)	0.104	0.043	1.109 (1.019–1.208)	0.017
	Age (2)	0.049	0.056	1.051 (0.941–1.173)	0.382
	Age (3)	−1.137	0.097	0.321 (0.265–0.388)	0.000
	Gender (1)	−0.478	0.039	0.620 (0.574–0.670)	0.000
	Household type (1)	−0.428	0.053	0.652 (0.588–0.723)	0.000
	Highest level of education (1)	−0.311	0.043	0.733 (0.674–0.797)	0.000
	Highest level of education (2)	−0.852	0.141	0.427 (0.324–0.563)	0.000
	Highest level of education (3)	−0.605	0.183	0.546 (0.382–0.782)	0.001
	Highest level of education (4)	−0.712	0.258	0.491 (0.296–0.814)	0.006
	Marital status (1)	−0.151	0.054	0.860 (0.773–0.956)	0.005
Model 2	Constant	0.665	0.092	1.945	0.000
	Age (1)	0.126	0.044	1.135 (1.041–1.237)	0.004
	Age (2)	0.106	0.058	1.111 (0.993–1.244)	0.067
	Age (3)	−1.025	0.099	0.359 (0.295–0.436)	0.000
	Gender (1)	−0.484	0.040	0.616 (0.570–0.667)	0.000
	Household type (1)	−0.375	0.054	0.687 (0.618–0.764)	0.000
	Highest level of education (1)	−0.324	0.043	0.723 (0.664–0.787)	0.000
	Highest level of education (2)	−0.859	0.142	0.424 (0.321–0.559)	0.000
	Highest level of education (3)	−0.616	0.183	0.540 (0.377–0.774)	0.001
	Highest level of education (4)	−0.723	0.259	0.485 (0.292–0.807)	0.005
	Marital status (1)	−0.175	0.054	0.839 (0.754–0.934)	0.001
	Physical activity participation	−0.334	0.086	1.397 (1.181–1.651)	0.000
	High-intensity activity	0.195	0.044	1.215 (1.116–1.323)	0.000
	Moderate-intensity activity	0.095	0.043	1.100 (1.012–1.196)	0.025
	Low-intensity activity	−0.188	0.062	0.829 (0.734–0.935)	0.002
	Social activity participation	−0.003	0.039	0.997 (0.924–1.076)	0.035
	Cognitive impairment	0.187	0.052	1.206 (1.089–1.335)	0.000

Significance tests are standardized: *p*<0.05 (95% Cl).

From these results, it can be concluded that among middle-aged and older adults (age ≥ 50 years) with chronic diseases, activity participation prevents the risk of depression, and physical activity is more effective than social activity. Research hypothesis 1 is valid. Additionally, the higher the cognitive level, the lower the risk of depression, confirming the validity of research hypothesis 2.

### 3.5 Multivariate logistic regression results

In the Model 3, the control category was Category A (no depression, assigned a value of “0”). In mild depression (Category B), physical activity participation [OR = 1.406; 95% CI (1.179–1.677)], high-intensity activity [OR = 1.248; 95% CI (1.145–1.361)], moderate-intensity activity [OR = 1.161; 95% CI (1.065–1.264)], and low-intensity activity [OR = 0.798; 95% CI (0.703–0.905)], with *p*-values less than 0.05, were significantly correlated with mild depression. In contrast, social activity participation and cognitive impairment were not significantly correlated with mild depression (*p* > 0.05). In moderate depression (Category C), high-intensity activity [OR = 1.353; 95% CI (1.207–1.518)], moderate-intensity activity [OR = 1.243; 95% CI (1.107–1.397)], low-intensity activity [OR = 0.713; 95% CI (0.607–0.837)], social activity participation [OR = 0.846; 95% CI (0.777–0.960)], and cognitive impairment [OR = 1.491; 95% CI (1.278–1.740)], with p-values less than 0.05, were significantly correlated with moderate depression. In contrast, there was no significant correlation between physical activity participation and moderate depression (*p* > 0.05). In major depression (Category D), moderate-intensity activity [OR = 1.543; 95% CI (1.077–2.209)], low-intensity activity [OR = 0.596; 95% CI (0.388–0.916)], social activity participation [OR = 0.692; 95% CI (0.502–0.954)], cognitive impairment [OR = 2.231; 95% CI (1.282–3.884)], with p-values less than 0.05, were significantly correlated with major depression. In contrast, physical activity participation and high-intensity activity were not significantly correlated with major depression (*p* > 0.05). The detailed parameters of Model 3 are presented in Table 8.

**Table 8:** Multivariate logistic regression results (Model 3, *N* = 12,767).

Depression level	Variable names	*β*	S.E.	OR (95% Cl)	*p-*value
Mild depression (Category B)	Constant	−0.192	0.076		0.011
	Physical activity participation	0.341	0.090	1.406 (1.179–1.677)	0.000
	High-intensity activity	0.222	0.044	1.248 (1.145–1.361)	0.000
	Moderate-intensity activity	0.149	0.044	1.161 (1.065–1.264)	0.001
	Low-intensity activity	−0.226	0.064	0.798 (0.703–0.905)	0.000
	Social activity participation	−0.023	0.040	0.977 (0.903–1.057)	0.564
	Cognitive impairment	0.057	0.053	1.058 (0.953–1.175)	0.288
Moderate depression (Category C)	Constant	−1.262	0.104		0.000
	Physical activity participation	0.174	0.116	1.190 (0.948–1.492)	0.134
	High-intensity activity	0.303	0.058	1.353 (1.207–1.518)	0.000
	Moderate-intensity activity	0.218	0.059	1.243 (1.107–1.397)	0.000
	Low-intensity activity	−0.338	0.082	0.713 (0.607–0.837)	0.000
	Social activity participation	−0.147	0.054	0.864 (0.777–0.960)	0.007
	Cognitive impairment	0.400	0.079	1.491 (1.278–1.740)	0.000
Major depression (Category D)	Constant	−4.011	0.344		0.000
	Physical activity participation	0.198	0.327	1.219 (0.642–2.313)	0.545
	High-intensity activity	0.203	0.173	1.225 (0.873–1.719)	0.240
	Moderate-intensity activity	0.434	0.183	1.543 (1.077–2.209)	0.018
	Low-intensity activity	−0.518	0.219	0.596 (0.388–0.916)	0.018
	Social activity participation	−0.369	0.164	0.692 (0.502–0.954)	0.024
	Cognitive impairment	0.802	0.283	2.231 (1.282–3.884)	0.005

The control category is category A (no depression, assignment “0”); The significance test criterion: *p* < 0.05 (95% Cl).

From these results, it can be concluded that among middle-aged and older adults (age ≥ 50 years) with chronic diseases, activity participation (physical activity and social activity) had a more significant effect on mild and moderate depression, while cognitive level had a more significant effect on moderate and severe depression, relative to no depression. Thus, research hypothesis 3 was supported.

## 4 Discussion

### 4.1 Principal findings

This study confirms that participation in activities (physical activity and social activity) prevents depression in middle-aged and older adults with chronic diseases. Both physical activity and social activity serve as non-pharmacological treatments to prevent depression and reduce depressive symptoms in this population. Physical activity was found to be more acceptable than social activity for middle-aged and older adults with chronic diseases, even when controlling for variables such as age, gender, household type, highest level of education, and marital status. Additionally, the study affirms that cognitive level is one of the factors influencing depression in middle-aged and older adults with chronic diseases. Specifically, middle-aged and older adults with chronic diseases who experience cognitive impairment are more likely to suffer from depression compared to those without cognitive impairment.

Depression among middle-aged and older adults with chronic diseases is a critical public health concern, linked to diminished quality of life, increased healthcare utilization, and heightened mortality. Physical activity is recognized as an affordable, side-effect-free, and feasible non-pharmacological adjunctive treatment to promote mental health, alleviate depressive symptoms, and enhance overall quality of life. Moreover, physical activity plays a crucial role in preventing depressive disorders, often demonstrating comparability or superiority to pharmacologic interventions (Lobelo et al., 2018). A meta-analysis study that included 15 studies comprising 191,130 participants and 2,110,588 person-years further confirmed the negative correlation between physical activity and depression, emphasizing that participation in physical activity can help reduce depressive symptoms (Pearce et al., 2022). Similarly, participation in social activities is identified as a significant means to prevent or alleviate depression, presenting itself as a valuable non-pharmacological adjunctive treatment. Various studies have underscored the protective role of engaging in valued social and religious activities against depression in older adults (Wilkinson et al., 2018). Social connectedness is recognized as a key element in depression prevention, with social disconnection, characterized by small social networks and infrequent social interactions, predicting heightened subsequent perceived social isolation (e.g., loneliness, lack of support) and, conversely, predicting increased depressive symptoms (Santini et al., 2020). Additionally, participation in social activities is noted to stimulate body systems and fortify lifelong attachment patterns, although the impact may vary based on the type of activity and cultural context (Lee and Kim, 2014). These findings are consistent with the conclusions of this paper, suggesting that participation in both physical activity and socialization has the potential to prevent or alleviate depression in middle-aged and older adults with chronic diseases.

Furthermore, this study reveals that among middle-aged and older adults with chronic diseases, participation in physical activities provides a more robust protective effect against depression than social activities. This difference may be due to the direct neurobiological benefits of exercise, such as increased brain-derived neurotrophic factor (BDNF) production, endorphin release, improved sleep quality, and reduced systemic inflammation, all of which are linked to better mental health. While social activities foster social support and reduce loneliness, their impact may be less direct. Despite controlling for various factors, residual confounding cannot be entirely ruled out. Therefore, this study suggests that prioritizing physical activity among interventions could be more effective in reducing the risk of depression in this population, thereby guiding clinicians and public health practitioners to adopt more targeted strategies. However, in terms of physical activity intensity, participation in moderate-intensity and vigorous-intensity activities may lead to depression in middle-aged and older adults with chronic illnesses. Physical activity induces neuroplastic processes associated with depression, diminishes inflammation, and enhances resistance to oxidative and physiological stress, thereby serving as a preventive and therapeutic measure against depression (Kandola et al., 2019). This effect could be attributed to the exacerbation of chronic disease symptoms in this population, leading to an increased likelihood of developing depression. However, participation in low-intensity physical activity can prevent depression in middle-aged and older adults with chronic diseases. It has been proposed that there exists a negative curvilinear dose–response relationship between physical activity and depression. The most significant benefits are observed when transitioning from no activity to some activity, with only minor additional benefits realized at higher levels of activity (Pearce et al., 2022).

For middle-aged and older adults grappling with chronic diseases, cognitive functioning emerges as a pivotal factor influencing their experience of depression. Those with cognitive impairment face an elevated likelihood of suffering from depression. In other words, middle-aged and older adults with lower cognitive functioning are more prone to experiencing depressive symptoms when confronted with chronic diseases, and these symptoms tend to be more severe. Some studies have indicated that middle-aged and older adults with chronic diseases exhibit poorer cognitive functioning and higher levels of depression, with depression showing a negative correlation with cognitive functioning (Chen et al., 2023; Jung et al., 2013). In contrast to prior research, the current study places greater emphasis on the role of cognitive impairment in depression among middle-aged and older adults with chronic diseases. It specifically delves into the impact of individual cognitive levels on the onset of depression in patients contending with chronic diseases. In clinical practice, comprehending the cognitive levels of middle-aged and older adults with chronic diseases holds significance for the prevention and intervention of depression. Enhancing patients’ cognitive levels can facilitate a better understanding and coping mechanism for chronic diseases, thereby reducing the negative emotions and psychological challenges stemming from these conditions. Furthermore, improvements in cognitive levels can promote active participation and cooperation with treatment, ultimately enhancing both the quality of life and treatment efficacy. For example, cognitive levels can be enhanced through the following approaches: regular mental exercises, participation in physical activities, maintaining social connections through community involvement, ensuring high-quality sleep, practicing mindfulness, and engaging in cognitive behavioral therapy.

The results of the multivariate logistic regression show that the association between cognitive level and the severity of depression, particularly moderate and severe depression, and the important impact of activity participation (including physical activity and socialization) on mild and moderate depression provide important insights into the management of depression in middle-aged and older adults with chronic conditions. Low levels of cognition may exacerbate moderate to severe depression by impairing basic functions such as memory, executive functioning, and attention, thereby weakening adaptive coping and psychological resilience. This decline exacerbates the sense of hopelessness that characterizes major depression, and thus targeted interventions for cognitive decline are needed in the treatment of more severe depression. In contrast, the therapeutic potential of physical and social activities in alleviating mild to moderate depression highlights the value of lifestyle interventions in the early stages of depression. The benefits of physical activity, such as enhanced neuroplasticity and reduced inflammation, coupled with the emotional support that comes with socialization, may serve as an effective strategy for preventing worsening of depressive symptoms. These findings advocate for a multifaceted approach to depression treatment that promotes activity engagement early on along with cognitive support for those facing late-stage depression, with the aim of tailoring interventions to the specific needs of this vulnerable population.

Finally, among middle-aged and older adults grappling with chronic diseases, regular participation in physical and social activities, including volunteer activities, demonstrates a protective effect against higher levels of depression (Rostant and Poggi, 2023). Furthermore, enhancing cognitive levels can serve as a preventative and interventional measure for depression. In conclusion, engaging in physical activity, social activity, and improving cognitive functioning can exert a positive clinical impact on preventing and intervening in depression among middle-aged and older adults with chronic diseases. This multifaceted approach can enhance mental health, improve quality of life, and provide robust evidence for clinical practice.

## 5 Limitations

This study examined the effects of activity participation and cognitive level on depression in middle-aged and older adults with chronic diseases, but there may be the following shortcomings: (1) this paper is a cross-sectional study, which limits the interpretation of the effects of activity participation and cognitive levels on depression in middle-aged and older adults with chronic diseases over time; (2) the study population of this paper comprises only middle-aged and older adults with chronic diseases in China, and the findings may differ from those in other countries with varying geographical and cultural distinctions; and (3) the factors controlled in this paper include only basic demographic variables, and there may be some sociological and physiological confounders that have not been incorporated into the control variables.

## 6 Conclusion

In middle-aged and older adults (age ≥ 50 years) with chronic diseases, activity participation and cognitive level play crucial roles in the prevention and intervention of depression. Physical activity, social engagement, and higher cognitive levels are protective factors against depression, while physical inactivity, lack of social participation, and cognitive impairment are risk factors that increase the likelihood of depression. Therefore, the policy-maker should strengthen depression prevention and treatment through policy guidance, resource investment, promotion of physical and social activities, cognitive enhancement, and the development of a comprehensive prevention and treatment system to safeguard the mental health of middle-aged and older adults with chronic diseases. Future research should consider conducting longitudinal studies with long-term follow-up surveys and clinical trials to explore the effects of various types of activity participation and cognitive levels on depression more deeply. Additionally, expanding the study to include a culturally diverse population of middle-aged and older adults with chronic illnesses and integrating potential confounders will help accurately assess the impact of activity participation and cognitive levels on depressive symptoms in this demographic.

## Data Availability

The original contributions presented in the study are included in the article/Supplementary material, further inquiries can be directed to the corresponding author.
